# Predicting of Ki-67 Expression Level Using Diffusion-Weighted and Synthetic Magnetic Resonance Imaging in Invasive Ductal Breast Cancer

**DOI:** 10.1155/2023/6746326

**Published:** 2023-04-05

**Authors:** Liying Zhang, Jisen Hao, Jia Guo, Xin Zhao, Xing Yin

**Affiliations:** Third Affiliated Hospital of Zhengzhou University, Department of Radiology, Zhengzhou, China

## Abstract

**Objectives:**

To investigate the association between quantitative parameters generated using synthetic magnetic resonance imaging (SyMRI) and diffusion-weighted imaging (DWI) and Ki-67 expression level in patients with invasive ductal breast cancer (IDC).

**Method:**

We retrospectively reviewed the records of patients with IDC who underwent SyMRI and DWI before treatment. Precontrast and postcontrast relaxation times (T1, longitudinal; T2, transverse), proton density (PD) parameters, and apparent diffusion coefficient (ADC) values were measured in breast lesions. Univariate and multivariate regression analyses were performed to screen for statistically significant variables to differentiate the high (≥30%) and low (<30%) Ki-67 expression groups. Their performance was evaluated by receiver operating characteristic (ROC) curve analysis.

**Results:**

We analyzed 97 patients. Multivariate regression analysis revealed that the high Ki-67 expression group (*n* = 57) had significantly higher parameters generated using SyMRI (pre-T1, *p*=0.001) and lower ADC values (*p*=0.036) compared with the low Ki-67 expression group (*n* = 40). Pre-T1 showed the best diagnostic performance for predicting the Ki-67 expression level in patients with invasive ductal breast cancer (areas under the ROC curve (AUC), 0.711; 95% confidence interval (CI), 0.609–0.813).

**Conclusions:**

Pre-T1 could be used to predict the pretreatment Ki-67 expression level in invasive ductal breast cancer.

## 1. Introduction

Breast cancer is a highly heterogeneous disease and the most frequently diagnosed cancer in almost all regions of the world [1, 2]. Because the various molecular subtypes of breast cancer lead to different clinical outcomes, molecular typing is critical for the accurate diagnosis and treatment of affected patients. According to the St. Gallen 2013 immunohistochemical (IHC) classification, IHC biomarkers, including estrogen receptor (ER), progesterone receptor (PR), human epidermal growth factor receptor 2 (HER2), and Ki-67 can be used as molecular subtypes in breast cancer [3].

Ki-67 is a nuclear DNA-binding protein highly expressed in the G1, S, and G2 phases of the cell cycle but not in the quiescent G0 phase. This protein is a clinically important proliferation marker used for grading various cancers [4, 5]. Clinical studies demonstrated that patients with a high Ki-67 index showed a favorable response to chemotherapy but had a relatively poor prognosis [4, 6]. Thus, timely identification of this population is important for appropriate treatment planning. Currently, we determine the preoperative Ki-67 proliferation index using IHC, which requires sufficient tumor tissue, typically obtained by core needle biopsy. However, biopsies are invasive and cannot be used to evaluate the status of the entire lesion or follow changes in the tumor microenvironment after neoadjuvant therapy [5, 7]. Furthermore, it is impossible to obtain tumor tissue specimens in many cases.

Magnetic resonance imaging (MRI) is a noninvasive tool that can assess multiple biomarkers in patients with breast cancer [8, 9]. The use of several advanced sequences in probing breast cancer biology, including diffusion-weighted imaging (DWI) and synthetic MRI (SyMRI) is still under investigation.

DWI is a noncontrast imaging technique that characterizes tissues by their random movement of water molecules within them [10–12]. The apparent diffusion coefficient (ADC) derived from DWI is a very promising quantitative parameter for the differential diagnosis of breast lesions and monitoring the effect of neoadjuvant treatment [13, 14]. However, while some studies analyzed associations between DWI and histopathological features in breast cancer, including between ADC and the expression of Ki-67, their findings were quite divergent. For example, some authors [5, 15] reported significant correlations between ADC and Ki-67 status, but others [16–18] did not identify this relationship.

SyMRI is a compilation pulse sequence that uses a multidynamic and multiecho (MDME) acquisition method. SyMRI provides absolute values for tissue properties, such as T1 and T2 relaxation times and proton density (PD), from a single acquisition with good accuracy and reproducibility, even across instruments from different vendors [19–23]. Thus far, some studies have reported on the value of SyMRI in discriminating breast lesions [24–27]. However, findings describing the relationships between the parameters generated using SyMRI and Ki-67 status are scarce and inconsistent.

Given the above facts, further assessment of these potential imaging biomarkers is warranted. Therefore, we investigated, for the first time, the ability of quantitative parameters derived from DWI and SyMRI to accurately predict the pretreatment Ki-67 expression level in invasive ductal breast cancer (IDC).

## 2. Material and Methods

### 2.1. Patients

**Figure 1:**
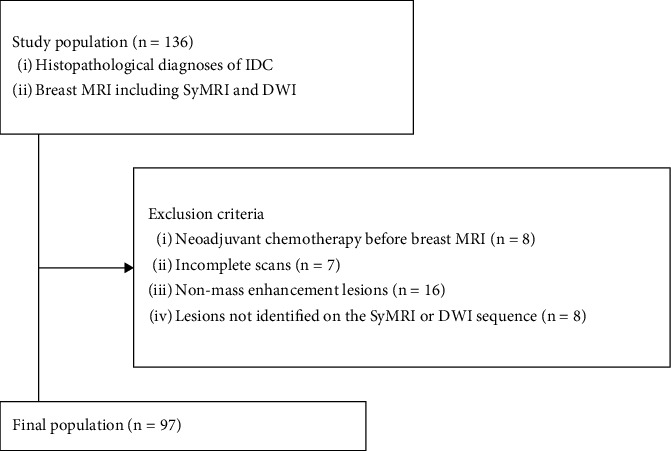
Flowchart of the selection of our study.

Our institutional review board approved this retrospective study and waived the requirement for informed consent. We enrolled consecutive female patients who underwent breast MRI (including SyMRI and DWI) for a breast lesion that was subsequently diagnosed as histopathological IDC after excision surgery or core needle biopsy between January 2020 and August 2022. The exclusion criteria were as follows: (1) neoadjuvant treatment before breast MRI, (2) incomplete scans, (3) nonmass enhancement lesions on dynamic contrast-enhanced MRI (DCE-MRI), and (4) lesions too small to be identified by the SyMRI or DWI sequence. For patients with multiple lesions, only the largest mass on the DCE-MRI was used for analysis. The patient selection is summarized in Figure 1.

### 2.2. MRI Protocol

All breast MR images were acquired using a 3T MRI scanner (Signa Pioneer, GE Healthcare, Chicago, IL, US) with an 8-channel phased-array breast coil. The patients entered the scanner feet first in the prone position. We used a power injector to administer a body weight-adjusted dose of Gd-DTPA (0.1 mmol/kg) intravenously at a rate of 3.0 ml/s to each patient, followed by 20 ml of saline flush at the same rate.

The scanning protocol consisted of conventional and quantitative scanning. T1- and T2-weighted images and DCE-MRI images were acquired. DCE-MRI was obtained using 3-dimensional (3D) differential subsampling with Cartesian ordering (DISCO) technology. Quantitative MR images included those obtained using SyMRI and DWI. SyMRI was performed before and after enhancement using a 2D fast spin-echo MDME sequence. The scan parameters are listed in Table 1.

### 2.3. Image Analysis

Two radiologists, each with more than seven years of breast MRI experience, performed a consensus review of the MRI findings. Lesion features, including size, shape, margin, enhancement pattern, and kinetic curves, were evaluated according to the Breast Imaging Reporting and Data System (BI-RADS) MRI lexicon.

Synthetic MRI data were postprocessed using SyMRI 8.0 software (Synthetic MR, Linköping, Sweden). Using DCE-MRI for lesion localization, a region of interest (ROI) was manually drawn to cover the enhancing solid portion of the lesion on approximately the same slice on the synthetic images and ADC maps. For each radiologist, the best quality image among the synthetic images was chosen for analysis, and values for T1, T2 relaxation time, and PD were simultaneously calculated within an ROI. All of the quantitative parameters (T1, T2, PD, and ADC values) were automatically produced in the workstation. The average measurements of the two radiologists were used for analysis. We recorded preparameters (pre-T1, pre-T2, and pre-PD) and postparameters (post-T1, post-T2, and post-PD generated from SyMRI.

### 2.4. Histopathologic Analysis

Pathologic reports were reviewed to identify lymph node metastasis and histologic grade. The histological grade was assessed using the Elston–Ellis system. ER, PR, and HER2 and the Ki-67 expression level were evaluated by IHC. ER and PR studies were considered positive when at least 1% of the tumor cells showed positive nuclear staining. The HER2 scanning intensity was scored as 0, 1+, 2+, or 3+; we considered 0 and 1+ negative, 2+ equivocal, and 3+ positive. An immunohistochemistry HER2 score of 2+ was further explored by in situ hybridization to determine HER2 gene amplification. A Ki-67 level ≥30% was considered high and a level <30% was considered low [28].

### 2.5. Statistical Analysis

Statistical analyses were performed using R language, version 4.1.0 (R Foundation for Statistical Computing, Vienna, Austria). The R packages doBy and ggplot2 were applied. All continuous variables are expressed as means ± standard deviation (SD), while categorical variables are shown as totals and proportions. The interobserver consistencies for all quantitative parameters between the two radiologists were evaluated with the intraclass correlation coefficient (ICC) analysis. Agreement was defined as good (ICC > 0.75), moderate (ICC = 0.5–0.75), or poor (ICC < 0.5). Clinicopathological and DCE-MRI features were compared using the Mann–Whitney *U* and chi-square tests. Quantitative parameters (T1, T2, PD, and ADC values) were performed both univariate and multivariate logistic regression analyses with a variable selection criterion of *p* < 0.05. We estimated the area under the receiver operating characteristic curve (AUC) to evaluate the predictive ability of the quantitative parameters. For all tests, *p* < 0.05 was considered statistically significant.

## 3. Results

### 3.1. Patients

We enrolled 136 patients. After applying our exclusion criteria, 97 patients (age range, 30–71 years; mean age, 49.5 years) were included in the study.

### 3.2. Clinicopathological and DCE-MRI Features

The Ki-67 proliferation indices obtained by analyses of core needle biopsies or surgically excised specimens ranged from 8% to 90%. We categorized 40 tumors (41.2%) as showing low proliferation (the low proliferation group) and the remaining 57 (58.8%) as demonstrating high proliferation (the high proliferation group) using the 30% cutoff. Our comparison of clinicopathological and DCE-MRI features between the two groups is shown in Table 2.

### 3.3. Interobserver Agreement on Quantitative Parameters

All ICCs between the two radiologists for the quantitative parameters generated from SyMRI and ADC maps were all greater than 0.75, indicating good agreements (range, 0.782–0.890; *p* < 0.05).

### 3.4. Quantitative Parameters

**Figure 2:**
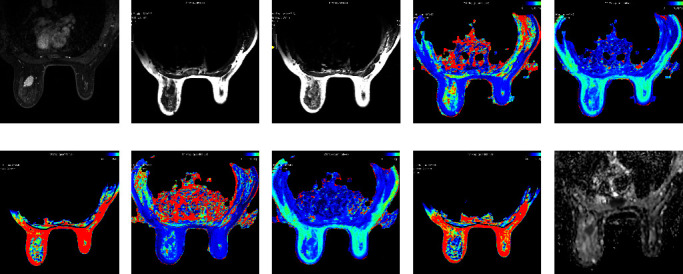
47-year-old female with invasive ductal carcinoma (IDC) of the left breast. Ki-67 expression was 20%. Axial contrast enhanced T1-weighted image (T1WI) showed an irregular homogeneous enhancing mass with circumscribed margin (a) The quantitative parameters were measured on SyMRI-T1WI before ((b) pre-T1 = 1495 ms, pre-T2 = 77 ms, and pre-PD = 80.8 pu) and after ((c) post-T1 = 683 ms, post-T2 = 69 ms, and post-PD = 67.4 pu) administration of the contrast medium. Similar images are shown for the mass on precontrast ((d) T1 mapping, (e) T2 mapping, and (f) PD mapping) and postcontrast ((g) T1 mapping, (h) T2 mapping, and (i) PD mapping) enhanced mapping images. The apparent diffusion coefficient (ADC) value was 0.936 × 10^−3^ mm^2^/s on the ADC map image (j).

**Figure 3:**
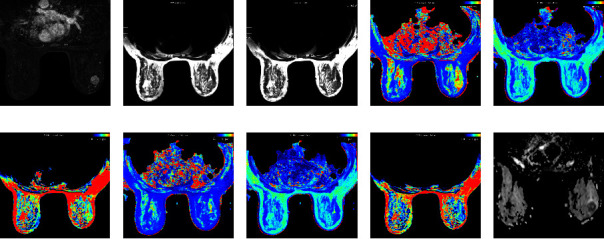
51-year-old female with invasive ductal carcinoma (IDC) of the right breast. Ki-67 expression was 50%. Axial contrast enhanced T1-weighted image (T1WI) showed an irregular heterogeneous enhancing mass with irregular margin (a) The quantitative parameters were measured on SyMRI-T2WI before ((b) pre-T1 = 1509 ms, pre-T2 = 80 ms, and pre-PD = 82 pu) and after ((c) post-T1 = 703 ms, post-T2 = 72 ms, and post-PD = 72.1 pu) administration of the contrast medium. Similar images are shown for the mass on precontrast ((d) T1 mapping, (e) T2 mapping, and (f) PD mapping) and postcontrast ((g) T1 mapping (h) T2 mapping, and (i) PD mapping) enhanced mapping images. The apparent diffusion coefficient (ADC) value was 0.763 × 10^−3^ mm^2^/s on the ADC map image (j).

**Figure 4:**
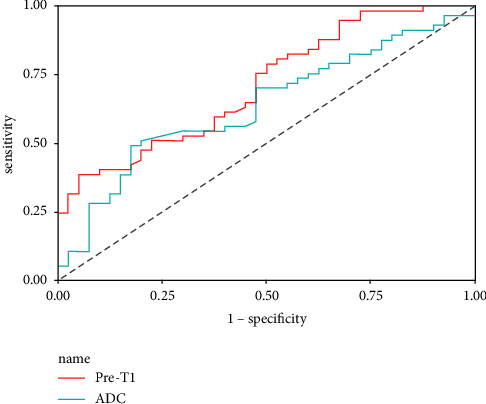
Receiver operating characteristic (ROC) curve of parameters for predicting the Ki-67 expression level. Pre-T1 showed a good diagnostic performance; its area under the curve was 0.711.

Histopathological and imaging findings of tumors and their associations with the quantitative parameters generated from SyMRI and DWI are summarized in Table 3. The post-T1 values were significantly lower in the noncircumscribed lesions than those in the circumscribed lesions (*p*=0.019). Furthermore, pre-T1, post-T1, and post-T2 in the high proliferation group were significantly higher than those in the low proliferation group (pre-T1, *p* < 0.001; post-T1, *p*=0.035; post-T2, *p*=0.009), and ADC values in the high proliferation group were significantly lower than those in the low proliferation group (*p*=0.022). Among these, multiple logistic regression analysis showed that pre-T1 (OR = 1.003; *p*=0.001) and ADC (OR = 0.035; *p*=0.036) values were statistically significant parameters in predicting the Ki-67 expression level (Table 4). Representative images from tumors with a low and high Ki-67 proliferation index are presented in Figures 2 and 3, respectively.  Table 5 shows the ROC analysis of the quantitative parameters. The area under the curve (AUC) of pre-T1 had the greatest discriminative ability with the highest AUC (AUC = 0.711; 95% confidence interval (CI), 0.609–0.813) (Figure 4).

## 4. Discussion

We compared multiple quantitative parameters (T1, T2, PD, and ADC values) to determine the expression level of Ki-67 in breast IDC. The results showed that the pre-T1 values were a significant predictor for the pretreatment Ki-67 expression level.

Matsuda et al. [29] reported that only the T1-Gd SD obtained from SyMRI was useful to predict Ki-67 status. However, their study only included patients with ER + breast cancer, and they used 14% as a cutoff value to define their low and high Ki-67 proliferation groups. According to the International Ki-67 in Breast Cancer Working Group (IKWG) Consensus Meeting Guideline (2019) [28], Ki-67 analysis should only be used to guide clinical decisions with 5% or less or 30% or more. Therefore, a reevaluation of the previous studies on associations between imaging parameters and Ki-67 expression was needed. We classified IDC lesions into two groups: low Ki-67 group (Ki-67 < 30%) and high Ki-67 group (Ki-67 ≥ 30%) in this study.

T1 values have been suggested to reflect extracellular expansion and underlying pathophysiological processes, using intrinsic and fundamental tissue property change [30, 31]. A previous research showed that hyperproliferative cancers (i.e., with high Ki-67 expression) might outgrow the oxygen supply of their vascular system, resulting in cell necrosis [32]. Therefore, we infer that there may be different native T1 values between the Ki-67 low and high groups. In the present study, tumors with high Ki-67 expression had higher native T1 values than those with low Ki-67 expression, and pre-T1 values differed significantly between the groups. The ROC analysis showed that a possible use of pre-T1 for discrimination of Ki-67 expression level in IDC has a relatively high AUC (AUC = 0.711). These results suggest the potential of pre-T1 values as a noninvasive surrogate biomarker for estimation of Ki-67 expression level.

Du et al. [33] showed that pre-T2 values had a statistically significant difference between the groups, and they used 14% as a Ki-67 cutoff value. In contrast to their results, we did not find a statistically significant association between pre-T2 values and Ki-67 expression level using 30% as a Ki-67 cutoff value.

Furthermore, our results showed that the post-T1 values were significantly lower in the noncircumscribed lesions than in the circumscribed lesions (*p*=0.019). Further studies are required to address the meaning of this result. Nevertheless, we did not find a correlation between the quantitative parameters generated from SyMRI after contrast agent injection and the Ki-67 expression level. This may be because our study did not include all the different molecular subtypes of breast cancer.

Another interesting aspect of this study is the fact that ADC values cannot be used as a surrogate marker for proliferation activity in IDC. Our results showed that ADC values in the high proliferation group were significantly lower than those in the low proliferation group (*p*=0.022). In spite of this, the ROC curve was not found to be useful in diagnostics (AUC = 0.683). The results are consistent with those reported previously [15, 34].

Our study had several limitations. First, patients were enrolled from a single institution with a limited number of relevant cases. Therefore, further studies with a large sample size are required to validate our findings. Second, patients with small lesions that could not be accurately located based on SyMRI were excluded from enrollment, possibly causing sampling bias. Third, we did not apply automatic image registration before and after enhancement to correct for the offset position between SyMRI and DWI, which could have reduced the stability of the analyzed data. Further study should be performed to confirm the accuracy of our findings.

## 5. Conclusions

In summary, synthetic MRI maybe a useful tool that can be used to predict the Ki-67 expression level in patients with IDC.

## Figures and Tables

**Table 1:** MRI sequences used in our study.

Sequence	T2WI	T1WI	DWI	DCE	SyMRI
Scan plane	Axial	Axial	Axial	Axial	Axial
TR (ms)	7400	790	3000	4.7	4000
TE (ms)	78	6.8	72	1.1/2.2	21/95
FA (°)	110	110	90	15	None
FOV (mm^2^)	360 × 390	360 × 390	360 × 390	360 × 390	360 × 390
Slice thickness (mm)	5	5	5	1.2	5
NEX	1.5	1	5	0.7	1
Fat saturation	Yes	None	Yes	Yes	None
*b* value (sec/mm^2^)	None	None	800	None	None
Scan time (min: s)	3 min	37 s	2 min 42 s	4 min 9 s	5 min 12 s

TR, repetition time; TE, echo time; FA, flip angle; FOV, field of view; NEX, number of excitations.

**Table 2:** Clinicopathological and DCE-MRI features of patients with IDC and low (<30%) or high (≥30%) Ki-67 proliferation.

Features	Ki-67 < 30%	Ki-67 ≥ 30%	*p* value
	(*n* = 40) (%)	(*n* = 57) (%)	
Age (year ± SD)	49.5 ± 9.0	49.3 ± 8.4	0.936
Lesion size (mm ±SD)	24.4 ± 10.8	28.1 ± 15.1	0.195
Shape			0.771
Oval/round	10 (25.0)	17 (29.8)	
Irregular	30 (75.0)	40 (70.2)	
Margin			0.352
Circumscribed	9 (22.5)	19 (33.3)	
Noncircumscribed	31 (77.5)	38 (66.7)	
LN metastasis			0.696
Positive	20 (50.0)	25 (43.9)	
Negative	20 (50.0)	32 (56.1)	
ER			0.225
Positive	32 (80.0)	38 (66.7)	
Negative	8 (20.0)	19 (33.3)	
PR			0.208
Positive	29 (72.5)	33 (57.9)	
Negative	11 (27.5)	24 (42.1)	
HER2			0.836
Positive	11 (27.5)	18 (31.6)	
Negative	29 (72.5)	39 (68.4)	
Histologic grade			0.173
Grade 1/2	36 (90.0)	44 (77.2)	
Grade 3	4 (10.0)	13 (22.8)	

DCE-MRI, dynamic contrast-enhanced magnetic resonance imaging; IDC, invasive ductal carcinoma; SD, standard deviation; ER, estrogen receptor; PR, progesterone receptor; HER2, human epidermal growth factor receptor 2; LN, lymph node.

**Table 3:** Histopathological and imaging findings of tumors and their associations with the quantitative parameters generated from SyMRI and DWI.

Parameters	Number (%)	Pre-T1(ms)	Pre-T2(ms)	Pre-PD(pu)	Post-T1(ms)	Post-T2(ms)	Post-PD(pu)	ADC (10^−3^ mm^2^/s)
Shape								
Oval/round	27	1750.4 ± 359.4	86.4 ± 12.2	77.6 ± 15.7	704.8 ± 165.0	73.2 ± 12.4	83..0 ± 16.3	0.934 ± 0.169
Irregular	70	1664.1 ± 386.2	83.2 ± 10.2	74.4 ± 13.0	647.7 ± 145.1	69.9 ± 8.9	80.0 ± 16.1	0.938 ± 0.162
*p* value		0.270	0.265	0.274	0.116	0.207	0.250	0.800
Margin								
Circumscribed	28	1732.4 ± 336.2	86.1 ± 12.0	76.8 ± 13.7	713.5 ± 153.7	73.7 ± 12.2	79.7 ± 13.3	0.908 ± 0.160
Noncircumscribed	69	1670.2 ± 396.1	83.2 ± 10.3	74.7 ± 13.9	643.4 ± 147.9	69.6 ± 8.8	81.3 ± 17.2	0.949 ± 0.164
*p* value		0.329	0.306	0.401	0.019	0.120	0.984	0.368
LN metastasis								
Positive	52	1663.8 ± 304.9	84.6 ± 10.6	75.7 ± 13.8	675.3 ± 153.4	70.8 ± 9.0	82.0 ± 15.3	0.938 ± 0.169
Negative	45	1716.2 ± 452.0	83.5 ± 11.3	74.8 ± 14.0	650.1 ± 151.4	70.8 ± 11.2	79.6 ± 17.2	0.936 ± 0.158
*p* value		0.789	0.839	0.590	0.487	0.871	0.356	0.879
Histologic grade								
Grade 1/2	80	1665.6 ± 349.6	83.9 ± 10.9	74.9 ± 14.1	658.3 ± 151.3	70.6 ± 10.5	81.7 ± 16.7	0.950 ± 0.150
Grade 3	17	1794.1 ± 494.4	85.0 ± 10.9	77.3 ± 12.5	688.9 ± 158.4	71.8 ± 7.5	77.0 ± 13.0	0.876 ± 0.209
*p* value		0.306	0.736	0.729	0.448	0.468	0.370	0.06
ER								
Positive	70	1665.9 ± 349.9	83.7 ± 11.0	75.4 ± 13.7	676.3 ± 155.2	70.5 ± 10.4	81.5 ± 15.5	0.916 ± 0.157
Negative	27	1745.9 ± 448.3	84.9 ± 10.7	75.1 ± 14.4	630.6 ± 141.6	71.5 ± 9.2	79.2 ± 15.5	0.992 ± 0.170
*p* value		0.754	0.435	0.760	0.183	0.444	0.363	0.037
PR								
Positive	62	1705.2 ± 411.7	83.8 ± 11.4	74.7 ± 14.7	670.4 ± 151.5	70.2 ± 10.4	81.7 ± 17.4	0.916 ± 0.157
Negative	35	1678.5 ± 362.5	84.6 ± 9.8	76.4 ± 12.0	651.6 ± 155.0	71.8 ± 9.4	79.4 ± 13.9	0.975 ± 0.169
*p* value		0.863	0.419	0.487	0.471	0.222	0.571	0.161
HER2								
Positive	29	1685.7 ± 407.5	86.8 ± 11.2	75.5 ± 13.9	676.2 ± 148.8	73.5 ± 11.1	79.7 ± 13.8	0.945 ± 0.176
Negative	68	1693.9 ± 308.9	82.9 ± 10.6	75.2 ± 13.9	658.2 ± 154.4	69.7 ± 9.4	81.4 ± 17.1	0.920 ± 0.131
*p* value		0.555	0.084	0.819	0.651	0.125	0.699	0.662
Ki-67 index								
Ki-67 < 30%	40	1516.2 ± 269.6	82.5 ± 12.1	71.6 ± 15.8	623.3 ± 114.4	67.9 ± 9.7	80.1 ± 17.4	0.976 ± 0.150
Ki-67 ≥ 30%	57	1808.8 ± 399.7	85.2 ± 9.8	77.9 ± 11.7	691.9 ± 169.2	72.9 ± 9.8	81.4 ± 15.4	0.910 ± 0.168
*p* value		<0.001	0.077	0.050	0.035	0.009	0.676	0.022
Molecular subtype								
Luminal A-like	17	1499.2 ± 303.9	80.9 ± 12.4	68.3 ± 15.9	599.4 ± 106.8	67..5 ± 10.3	78.7 ± 20.1	0.924 ± 0.140
Luminal B-like	54	1722.5 ± 346.8	84.7 ± 10.3	77.0 ± 13.0	698.7 ± 160.3	71.4 ± 10.2	82.1 ± 15.3	0.914 ± 0.162
HER2+	13	1630.7 ± 340.6	84.5 ± 10.7	75.2 ± 12.9	620.4 ± 94.5	70.5 ± 7.8	79.4 ± 16.2	0.936 ± 0.109
Triple-negative	13	1849.7 ± 539.9	85.1 ± 11.5	77.4 ± 14.0	645.2 ± 184.2	72.8 ± 10.8	80.1 ± 15.3	1.050 ± 0.209
*p* value		0.183	0.518	0.199	0.088	0.389	0.815	0.134

SyMRI, synthetic magnetic resonance imaging; ADC, apparent diffusion coefficient; PD, proton density.

**Table 4:** Multivariate logistic regression analysis of variables associated with Ki-67 index.

Variables	Multivariate logistic regression		
	OR	(95% CI)	*p* value
Pre-T1	1.003	1.001–1.004	0.001
Post-T1	1.002	0.998–1.006	0.366
Post-T2	1.031	0.974–1.102	0.324
ADC	0.035	0.295–0.688	0.036

ADC, apparent diffusion coefficient; OR, odds ratio; CI, confidence interval.

**Table 5:** Diagnostic value of parameters in the low (<30%) or high (≥30%) Ki-67 proliferation groups.

Parameters	Sensitivity (%)	Specificity (%)	AUC	*p* value	95% CI
Pre-T1	38.6	95.0	0.711	<0.001	0.609–0.813
ADC	48.1	82.5	0.637	0.016	0.533–0.732

ADC, apparent diffusion coefficient; AUC, area under curve; CI, confidence interval.

## Data Availability

All data generated or analyzed during this study are included in this article.
